# Association of Electronegative LDL with Macrophage Foam Cell Formation and CD11c Expression in Rheumatoid Arthritis Patients

**DOI:** 10.3390/ijms21165883

**Published:** 2020-08-16

**Authors:** Ching-Kun Chang, Po-Ku Chen, Joung-Liang Lan, Shih-Hsin Chang, Tsu-Yi Hsieh, Pei-Jyuan Liao, Chu-Huang Chen, Der-Yuan Chen

**Affiliations:** 1 Rheumatology and Immunology Center, China Medical University Hospital, Taichung 404, Taiwan; kun80445@gmail.com (C.-K.C.); pago99999@gmail.com (P.-K.C.); pagey06@yahoo.com.tw (P.-J.L.); 2 Translational Medicine Laboratory, China Medical University Hospital, Taichung 404, Taiwan; jounglan@me.com (J.-L.L.); sherry61976@hotmail.com (S.-H.C.); 3 College of Medicine, China Medical University, Taichung 404, Taiwan; 4 Rheumatic Diseases Research Center, China Medical University Hospital, Taichung 404, Taiwan; 5 Ph.D. Program in Translational Medicine and Rong Hsing Research Center for Translational Medicine, National Chung Hsing University, Taichung 402, Taiwan; 6 Department of Medical Education and Research, Taichung Veterans General Hospital, Taichung 407, Taiwan; zuyihsieh@gmail.com; 7 Vascular and Medicinal Research, Texas Heart Institute, Houston, TX 6770, USA; cchen@texasheart.org; 8 Institute for Biomedical Sciences, Shinshu University, Nagano 390-8621, Japan

**Keywords:** L5, macrophage foam cell, CD11c expression, atherosclerosis, rheumatoid arthritis (RA)

## Abstract

L5, the most negatively charged subfraction of low-density lipoprotein (LDL), is implicated in atherogenesis, but the pathogenic association is relatively unexplored in patients with rheumatoid arthritis (RA). We examined the role of L5 LDL in macrophage foam cell formation and the association of L5 with CD11c expression in THP-1 cells and RA patients. Using quantitative real-time PCR, we determined mRNA expression levels of *ITGAX*, the gene for CD11c, a marker associated with vascular plaque formation and M1 macrophages in atherogenesis, in 93 RA patients. We also examined CD11c expression on THP-1 cells treated with L5 by flow cytometry analysis and the plasma levels of inflammatory mediators using a magnetic bead array. We found a dose-dependent upregulation of foam cell formation of macrophages after L5 treatment (mean ± SEM, 12.05 ± 2.35% in L5 (10 µg/mL); 50.13 ± 3.9% in L5 (25 µg/mL); 90.69 ± 1.82% in L5 (50 µg/mL), *p* < 0.01). Significantly higher levels of CD11c expression were observed in 30 patients with a high percentage of L5 in LDL (L5%) (0.0752 ± 0.0139-fold) compared to 63 patients with normal L5% (0.0446 ± 0.0054-fold, *p* < 0.05). CD11c expression levels were increased in the L5-treated group (30.00 ± 3.13% in L5 (10 µg/mL); 41.46 ± 2.77% in L5 (50 µg/mL), *p* < 0.05) and were positively correlated with plasma levels of interleukin (IL)-6 and IL-8. L5 augmented the expression of IL-6, IL-8, and tumor necrosis factor-α (TNF-α) on monocytes and macrophages. Our findings suggest that L5 may promote atherogenesis by augmenting macrophage foam cell formation, upregulating CD11c expression, and enhancing the expression levels of atherosclerosis-related mediators.

## 1. Introduction

Atherosclerosis, a chronic inflammatory process, is characterized by atheromatous plaque buildup and associated with increased cardiovascular disease (CVD) risk [1]. One of the first events in atherosclerosis is the formation of macrophage foam cells caused by the oxidative modification of low-density lipoprotein (LDL) [2]. Rheumatoid arthritis (RA) is an inflammatory articular disease [3] complicated by accelerated atherosclerosis and elevated CVD risk [4,5]. The high CVD burden in RA patients would be explained by traditional CV risk factors and systemic inflammation in this disease [6,7]. Therefore, inflammatory mediators such as interleukin (IL)-6, IL-8, and tumor necrosis factor-α (TNF-α) are commonly involved in the pathogenesis of RA-related atherosclerosis [8,9,10,11,12]. 

Because RA patients have low low-density lipoprotein cholesterol (LDL-C) levels combined with an elevated CVD risk (a lipid paradox) [13], LDL-C probably contains critical atherogenic components not reflected in the absolute LDL-C concentration. Anion-cation exchange can be used to divide LDL-C into L1-L5 subfractions, of which L5 is the most negatively charged. Increasing evidence indicates a vital role for L5 in the pathogenesis of atherosclerosis [14]. We recently reported that an elevated the percentage of L5 in LDL (L5%) is associated with an increased CVD risk in patients with RA or systemic lupus erythematosus [15,16]. Therefore, it is important to study the role of L5 in foam cell formation in macrophages. 

Accumulating evidence indicates that variants in genes such as *ABCA1* (ATP-binding cassette, sub-family A, member 1) and *NPC1* (Niemann-Pick disease, type C1) are related to CVD risk [17,18,19,20]. CD11c, a membrane protein that is associated with β2 integrins, is encoded by the gene *ITGAX (Integrin Subunit Alpha X)* located on chromosome 16p11.2. Previous studies revealed a higher expression of CD11c on circulating monocytes from RA patients than on monocytes from healthy control (HC) subjects [21]. CD11c is a cell surface protein that participates in cell adhesion [22], and knocking out its gene (*ITGAX)* in mice decreased vascular plaque formation [23]. These observations suggest a pathogenic role of CD11c expression in atherogenesis. However, the relationship between electronegative L5 and CD11c expression in the development of atherogenesis in RA is not clear. 

In this pilot study, we aimed to investigate the role of LDL-L5 in macrophage foam cell formation and the association of L5 with CD11c expression in THP-1 cells and in RA patients. We also evaluated the correlation between CD11c expression and plasma levels of inflammatory mediators and validated L5’s pathogenic role in this process in an in vitro cell-based assay.

## 2. Results

### 2.1. Clinical Characteristics of RA Patients

Of the 93 RA patients, 66 (71.0%) tested positive for rheumatoid factor (RF) and 63 (67.7%) for anti-citrullinated peptide antibodies (ACPA). Significantly higher levels of C-reactive protein were observed in RA patients with high L5% compared to those with normal L5% (*p* < 0.05) (Table 1). We found no significant differences between RA patients with high L5% and normal L5% in demographic variables, clinical characteristics, the proportion of positivity for RF or ACPA, disease activity scores, the proportion of comorbidities, or medication use.

### 2.2. Comparison of Lipid Profiles, QRISK-2 Scores, and CVD Events between RA Patients with High L5% and Normal L5%

As illustrated in Table 1, RA patients with high L5% had significantly greater QRISK-2 scores, a global 10-year CVD risk score, than in those with normal L5% (*p* < 0.01). During the two-year follow-up period, a higher rate of CVD events was observed in RA patients with high L5% (6/30, 20.0%) compared to those with normal L5% (4/63, 6.3%, *p* = 0.071). However, there were no significant differences in lipid profile including total cholesterol (TC), high-density lipoprotein-cholesterol (HDL-C), triglyceride, or low-density lipoprotein-cholesterol (LDL-C) between RA patients with high L5% and with normal L5%.

### 2.3. The Effects of L5 on Macrophage Foam Cell Formation

**Figure 1:**
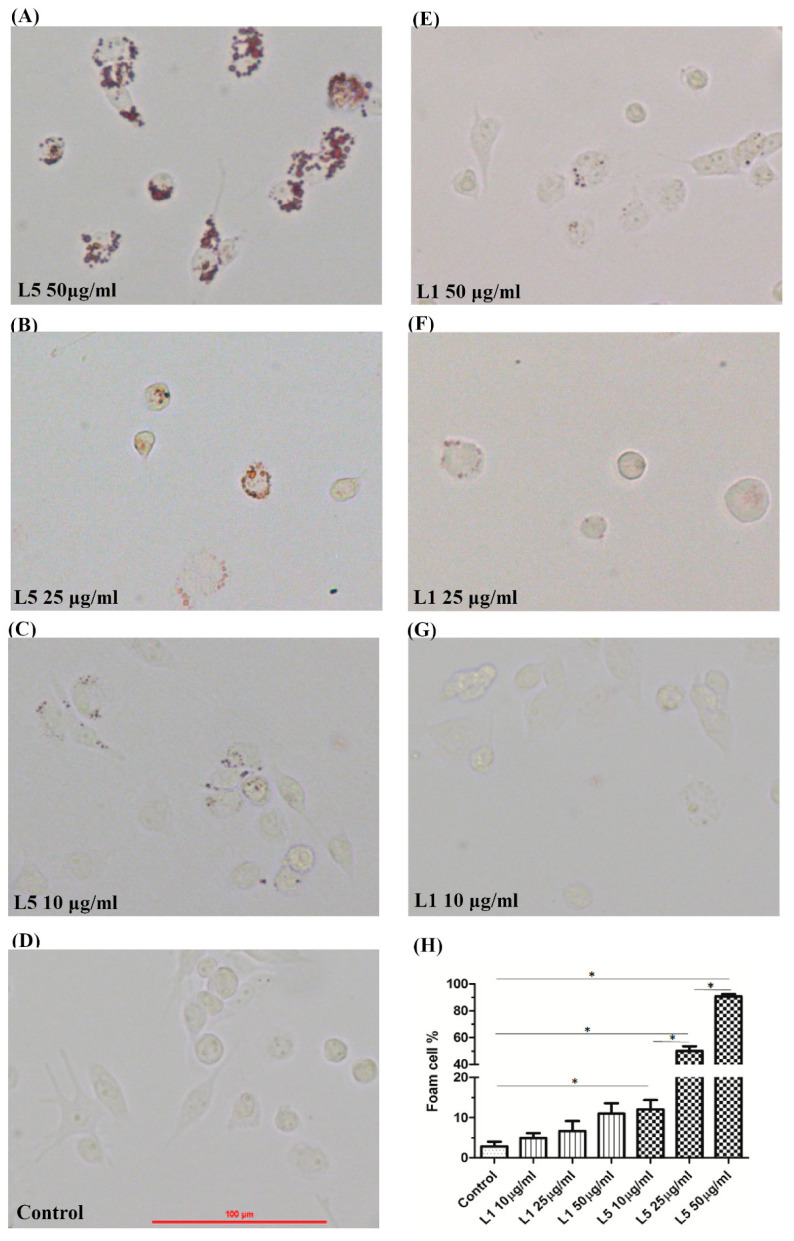
Effects of L5 on macrophage foam cell formation. THP-1 cells were incubated with (**A**) high-dose L5 (50 μg/mL), (**B**) midden-dose L5 (25 μg/mL), (**C**) low-dose L5 (10 μg/mL), (**D**) control (phosphate buffered saline, (**E**) high-dose L1 (50 μg/mL), (**F**) midden-dose L1 (25 μg/mL), and (**G**) low-dose L1 (10 μg/mL) for 48 h. (**H**) Difference in the proportion of macrophage foam cell formation among the different groups. Data are presented as the mean ± SEM for three independent experiments. * *p* < 0.05, determined by one-way ANOVA.

To investigate the potential effects of L5 on macrophage foam cell formation, we treated THP-1 cells with 10 ng/ml phorbol myristate acetate (PMA) for 48 h to stimulate differentiation into macrophages. Then, the monocyte-derived macrophages were stimulated with different doses of L5 (10, 25, or 50 μg/mL) or L1 (10, 25, or 50 μg/mL) at 37 °C for 48 h. As illustrated in Figure 1, the L5 induced foam cell formation, and a dose-dependent upregulation of foam cell formation of macrophages after L5 treatment (mean ± standard error of mean (SEM), 12.05 ± 2.35% in L5 (10 µg/mL); 50.13 ± 3.9% in L5 (25 µg/mL); 90.69 ± 1.82% in L5 (50 µg/mL), *p* < 0.05). The high-dose L5 also induced significantly more foam cell formation than high-dose L1 (11.00 ± 2.59%, *p* < 0.05). 

### 2.4. Comparison of CD11c mRNA Expression Levels between RA Patients with High L5% and Normal L5%

To identify the genes potentially involved in RA-related atherosclerosis, we examined the mRNA expression of 20 candidate genes. The results showed 10 differentially expressed genes in RA patients compared to healthy controls (Figure S1). Given the augmented effects of L5 on macrophage foam cell formation, we identified the candidate genes involved in L5-related atherosclerosis in RA patients by quantitative real-time polymerase chain reaction (qRT-PCR) assay. The results showed a significant difference in the expression levels of 10 candidate genes: *ABCA1, ACTR2, AFF4, CD11c, NPC1, PPFIA1, SMARCA2, WSB1, ZFAND6,* and *ZNF652*, between RA patients and healthy controls (Figure S1). Then, we examined the difference in the expression levels of these 10 genes between RA patients with high L5% and normal L5%. The results indicated a significant difference in the mRNA expression levels of only one gene, *ITGAX* (for CD11c), between the two groups (Figure S2).

**Figure 2:**
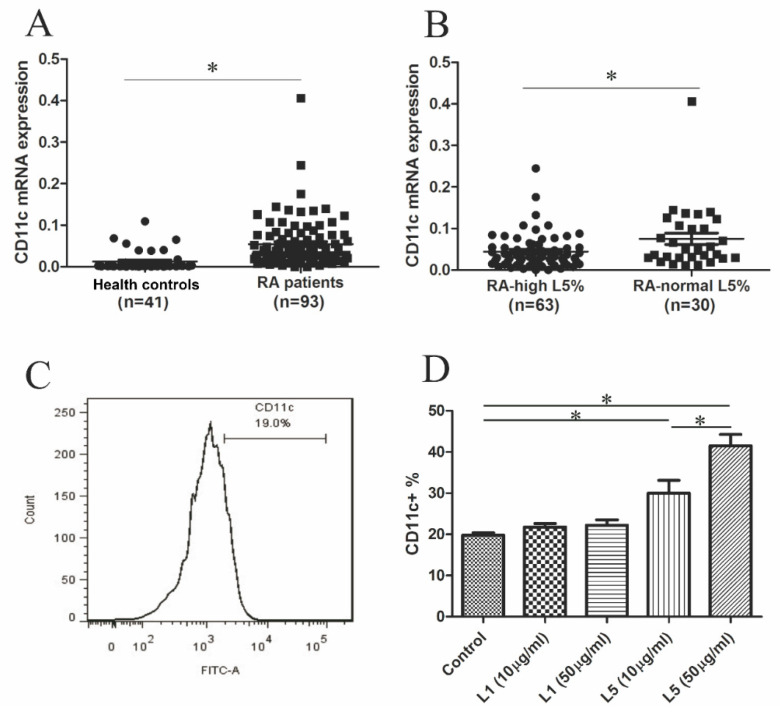
Comparison of CD11c (*ITGAX*) mRNA expression (**A**) between rheumatoid arthritis (RA) patients and healthy controls (HC), and (**B**) between RA patients with high L5% and normal L5%. (**C**) Representative histogram of the flow cytometric analysis of CD11c expression on THP-1 cells. (**D**) Bar graph showing the percent CD11c expression levels on THP-1 cells treated with different doses of L1 or L5 and fetal bovine serum (FBS)-treated control cells. Data are the mean ± SEM for three independent experiments. * *p* < 0.05.

In enrolled participants, we examined the difference in CD11c mRNA expression levels between RA patients (*n* = 93) and healthy controls (*n* = 41). The results showed significantly higher levels of CD11c expression in RA patients (relative of actin expression, mean ± SEM, 0.0545 ± 0.0059 folds) compared to healthy controls (0.0126 ± 0.0037 folds, *p* < 0.01, Figure 2A). Moreover, significantly higher levels of CD11c expression were observed in RA patients with high L5% (0.0752 ± 0.0139 folds) than in those with normal L5% (0.0446 ± 0.0054 folds, *p* < 0.05, Figure 2B). After exclusion of patients with cardiovascular events, we still revealed a significant difference in the CD11c expression levels between patients with high L5% (mean ± SEM, 0.0617 ± 0.0093 folds) and normal L5% (0.0404 ± 0.0051 folds, *p* < 0.05).

### 2.5. The Effects of L5 on CD11c Expression in THP-1 Cell

Given the higher levels of CD11c expression in patients with high L5% compared to those with normal L5%, we examined whether L5 could induce CD11c expression on THP-1 cells. After a 48-h stimulation with different doses of L5 or L1, we analyzed CD11c expression by flow cytometry analysis (Figure 2C). CD11c expression levels were increased in L5-treated cells (mean ± SEM, 30.00 ± 3.13% in L5 10 µg/mL; 41.46 ± 2.77% in L5 50 µg/mL, *p* < 0.05); the increases were significantly higher than those in L1-treated (21.77 ± 0.83% in L1 10 µg/mL; 22.20 ± 1.28% in L1 50 µg/mL, both *p* < 0.05) or untreated cells (19.82 ± 0.57%, *p* < 0.05, Figure 2D).

### 2.6. Correlation between CD11c Expression Levels and Plasma Levels of Inflammatory Mediators in RA Patients

Because CD11c is a marker for classically activated macrophages (M1 macrophages) [24,25], which produce proinflammatory cytokines [26], we examined the correlation between CD11c expression and plasma levels of inflammatory mediators in RA patients. As shown in Figure S3, CD11c expression levels were positively correlated with plasma levels of IL-6 (*r* = 0.2928, *p* = 0.0352) or IL-8 (*r* = 0.2917, *p* = 0.0359). There was no significant correlation between CD11c expression levels and other inflammatory mediators.

### 2.7. The Effects of L5 on Cytokine Expression in THP-1 Cells

**Figure 3:**
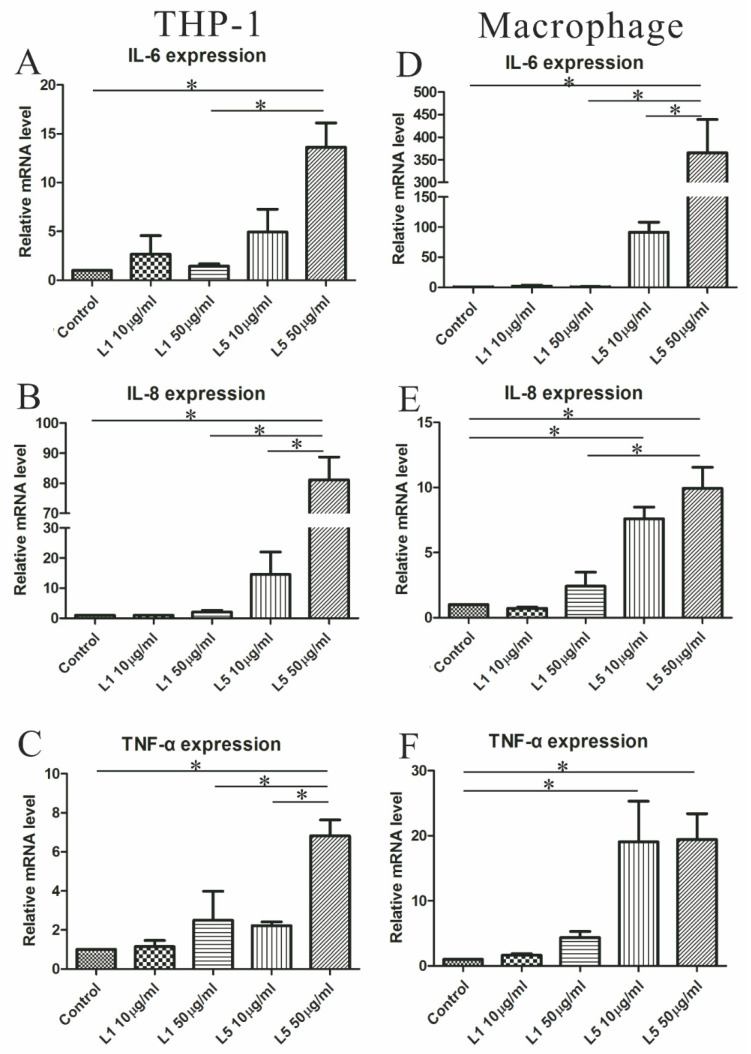
Effects of L5 on the expression of cytokines in monocytes and macrophages. The differences in the mRNA expression levels are shown for IL-6 (**A**), IL-8 (**B**), and (**C**) tumor necrosis factor-α (TNF-α) in THP-1 cells (human monocytic cell line) treated with different doses of L1 and L5. The differences in the mRNA expression levels are shown for IL-6 (**D**), IL-8 (**E**), and (**F**) TNF-α in THP-1 cells-derived macrophages treated with different doses of L1 and L5. Data are the mean ± SEM for three independent experiments. * *p* < 0.05.

Because of the significant association of L5 with the expression of CD11c, which affects secretion of inflammatory mediators, we examined whether L5 had an effect on the expression of proinflammatory cytokines, such as IL-6, IL-8, and TNF-α, in THP-1 cells and THP-1 cell-derived macrophages. The results showed that L5 upregulated the expression of IL-6, IL-8, and TNF-α in both monocytes and macrophages (Figure 3A–F). Moreover, the levels of IL-6 and IL-8 were significantly higher in monocytes or macrophages treated with high-dose L5 (relative of control group, mean ± SEM, 13.6 ± 2.5 folds, 81.1 ± 7.6 folds; and 365.9 ± 73.5 folds, 9.9 ± 1.6 folds, respectively) than in those treated with high-dose L1 (1.4 ± 0.2 folds, 2.1 ± 0.6 folds; and 0.9 ± 0.4 folds, 2.4 ± 1.1 folds, respectively, all *p* < 0.05, Figure 3A,B,D,E). Monocytes and macrophages treated with high-dose L5 (relative of control group, mean ± SEM, 6.82 ± 0.82 folds, 19.45 ± 3.93 folds) also induced significantly higher TNF-α expression than that seen in untreated cells (all *p* < 0.05, Figure 3C,F).

### 2.8. Proposed Model for the Potential Role of L5 in RA-Related Atherogenesis

**Figure 4:**
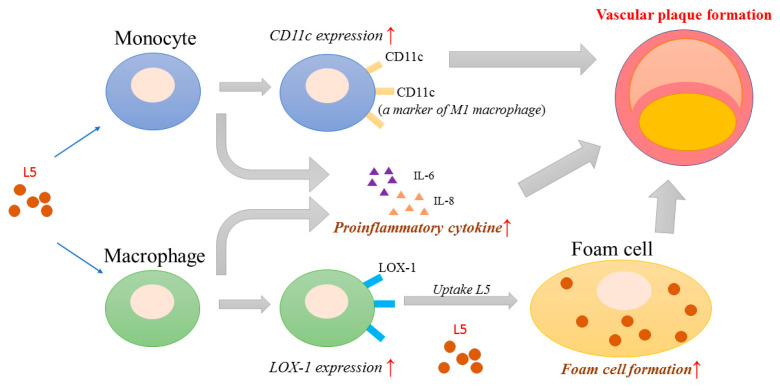
Proposed model for the potential role of L5 in RA-related atherogenesis. L5 induces the expression of CD11c, which is associated with vascular plaque formation [23,27] and is a marker for M1 macrophages that secrete proinflammatory cytokines [26]. L5 also upregulates the expression of inflammatory cytokines, interleukin (IL)-6 and IL-8, in both monocytes and macrophages. In addition, L5 induces LOX-1 expression and promotes foam cell formation by uptake of L5. The elevated levels of inflammatory cytokines and macrophage foam cell formation may contribute to vascular plaque formation in RA-related atherogenesis.

L5 induces the expression of CD11c, which has been reported to be associated with vascular plaque formation [23,27] and as a marker of M1 macrophages with secretion of inflammatory cytokines [26]. L5 also upregulates the expression of IL-6 and IL-8 on both monocytes and macrophages. We have previously shown that L5 induces the expression of lectin-like oxidized LDL receptor-1 (LOX-1) [15], a receptor that is involved in a variety of atherogenic responses including foam cell formation [16]. The combination of foam cell accumulation and increased cytokine activity in the microenvironment synergistically promotes plaque formation (Figure 4).

## 3. Discussion

The association between plasma L5 and the increased CVD risk in RA provides a new explanation for the paradoxically normal plasma LDL levels seen in these patients [15]. The present study was designed to explore the underlying molecular mechanisms of this unique clinical phenomenon. Here, we have shown that treatment with L5 upregulated foam cell formation in THP-1-derived-macrophages, whereas L1, even at high doses, exerted no effect. To identify the genetic variants attributable to L5-related atherosclerosis, we compared expression levels of candidate genes in RA patients with high L5% and normal L5%. We found a significant difference in the expression of *ITGAX*, the gene that encodes the integrin CD11c, which plays a pivotal role in vascular plaque formation [23]. L5 induced CD11c expression in THP-1 cells. Moreover, CD11c expression levels were positively correlated with plasma levels of IL-6 and IL-8. L5 also upregulated the expression of IL-6, IL-8, and TNF-α in in vitro assays. These findings suggest that L5 may contribute to atherogenesis by promoting foam cell formation, upregulating CD11c expression, and inducing the secretion of atherosclerosis-related mediators (Figure 4).

Dyslipidemia is a well-established traditional risk factor for atherosclerosis [6,7], and foam cell formation occurs in the early stage of atherogenesis in RA patients [28,29]. In the present study, we are the first to show significant enhancement of foam cell formation in macrophages treated with L5, which may be related to higher QRISK-2 scores in our patients with high L5% compared to those with normal L5%. Recent studies reveal that L5 promotes the differentiation of monocytes into macrophages in a dose-dependent manner [30], and L5 containing glycosylated apolipoprotein(apo) E may contribute to atherogenicity [31]. In addition, L5 containing apoCIII has been reported to induce monocytes adhesion with endothelial cells to contribute atherogenesis [32]. These findings support the finding that plasma L5% was significantly higher in RA patients with subclinical atherosclerosis than in those without [15], and support the link between L5 and the CVD risk [15,33,34]; L5 promotes foam cell formation.

The increased CVD risk in RA patients results from the intricate interactions among traditional CV risk factors, systemic inflammation, and genetic components [6,7,28,35]. In accordance with the findings that L5 is closely related to an increased CVD risk in autoimmune diseases [15,16], RA patients with high L5% in the current study had significantly higher levels of CD11c mRNA expression compared to those with normal L5%. To validate L5’s association with CD11c expression, we examined the effects of L5 on CD11c expression in THP-1 cells using flow cytometry analysis. The results showed that L5 enhanced CD11c expression, whereas L1 had no effect. The different effects may be related to the varied composition of apolipoprotein(apo) in L1, and L5:L1 contains apoB100, while L5 contains apoAI, apoE, and apoCIII [36]. Given the significant association of CD11c expression with vascular plaque formation in atherosclerosis in mice [23] or patients [27], this finding strongly supports the atherogenic role of L5 in RA and possibly other autoimmune diseases. 

CD11c is a probable marker of proinflammatory M1 macrophages, which have the propensity to secrete inflammatory mediators [26,37] and thus promote atherogenesis [24,27]. Both IL-6 and IL-8 are well-established mediators of RA-related atherosclerosis [8,9,10], and we found that CD11c expression was positively correlated with increasing levels of IL-6 and IL-8. This prompted us to examine the effects of L5 on the expression of atherosclerosis-related mediators in monocytes and macrophages. Our finding that L5 upregulated the expression levels of IL-6, IL-8, and TNF-α in monocytes and macrophages further substantiated L5’s atherogenic role through a mechanism mediated by CD11c.

Despite the novel findings in this pilot study, there were still some limitations. First, the sample size of RA patients in whom we could observe the emergence of CVD was small, which may reduce the statistical power. The effects of other medications, such as corticosteroids and disease-modifying anti-rheumatic drugs (DMARDs), should be considered because they may affect plasma levels of lipids and inflammatory mediators [38]. Finally, none of the enrolled patients in our study were in the early RA stage, which may limit the generalizability of these results to the whole population.

## 4. Materials and Methods

### 4.1. Study Population

In this prospective study, we enrolled 93 patients who met the 2010 revised criteria of the American College of Rheumatology for RA [39] and who had an active disease status. Disease activity was assessed by using the 28-joint disease activity score (DAS28) [40], and active status was defined as DAS28 ≥ 3.2. Each patient had previously received corticosteroids, nonsteroidal anti-inflammatory drugs, and at least one conventional synthetic disease-modifying anti-rheumatic drug (csDMARD). Patients with a recent history (i.e., within one year before enrollment) of coronary artery disease or ischemic stroke were excluded. Follow-up for the emergence of CVD, which included acute myocardial infarction and ischemic stroke, was done for at least two years. The healthy control (HC) group comprised 41 sex- and age-matched healthy volunteers with no rheumatic disease. The Institutional Review Board of our hospital approved this study (CMUH107-REC2-038, approval date 19 March 2018), and each participant’s written consent was obtained according to the Declaration of Helsinki.

### 4.2. Determination of Plasma Lipid Profiles and Atherogenic Index (AI)

All blood samples were collected from patients in the early morning after an overnight fast for 12 h. Plasma levels of TC, triglyceride, HDL-C, and LDL-C were measured by using enzymatic methods with a chemistry analyzer (Hitachi 7600, Hitachi, Tokyo, Japan) according to the manufacturer’s instructions. The AI (i.e., the ratio of TC /HDL-C) was calculated.

### 4.3. Measurement of 10-Year Risk of CVD Including QRISK-2 Score

Global 10-year risk for a heart attack or stroke was estimated by calculating the QRISK-2 scores [41] on the website: https://www.qrisk.org. Briefly, factors including age, sex, ethnicity, physical characteristics, total cholesterol/HDL-C ratio, self-reported smoking status, diabetic status, the presence of chronic kidney disease, and family history of heart disease were considered in determining the QRISK-2 score.

### 4.4. Isolation and Fractionation of LDL-C

Lipoproteins were isolated with sequential potassium bromide density ultracentrifugation as described [42]. The plasma was obtained from freshly collected whole blood samples, and 1% antibiotics (penicillin/streptomycin stock solution, Gibco), 0.5 mM EDTA (Thermo Fisher Scientific, Waltham, MA, USA), and a protease inhibitor cocktail (cOmplete, Roche, Sigma-Aldrich, St. Louis, MO, USA) were added to avoid ex vivo oxidation and degradation. LDL-C particles were isolated by using sequential potassium bromide density ultracentrifugation. Purified LDL-C was dialyzed against a degassed solution of 20 mM Tris-HCl and 0.5 mM EDTA at 4 °C with five buffer changes (once/day).

### 4.5. Anion-Exchange Chromatography Purification of LDL-C Subfractions

LDL subfractions were separated by using UnoQ12 anion-exchange columns (Bio-Rad Laboratories, Inc., Hercules, CA, USA) with an NGC Quest 10 chromatography system (Bio-Rad). The columns were pre-equilibrated with buffer A (0.02 M Tris-HCl (pH 8.0) and 0.5 mM EDTA) in a 4 °C cold room. After dialysis with buffer A, 100 mg of LDL in 10 mL was injected onto a UnoQ12 column and eluted at a flow rate of 2 mL/min with a multistep gradient of buffer B (1 M NaCl in buffer A). Five LDL fractions were eluted with a multistep gradient of buffer B according to electronegativity. L1 was the effluent collected between fractions 11 to 14 (18–28 min); L2, fractions 15 to 16 (28–32 min); L3, fractions 17 to 24 (32–48 min); L4, fractions 25 to 30 (48–60 min); and L5, fractions 31 to 40 (60–80 min). Protein concentrations were determined by using the bicinchoninic acid method. The respective fractions were then concentrated with Centriprep filters (YM-30, MilliporeSigma, Burlington, MA, USA) and sterilized by passage through 0.22 μm syringe filters.

### 4.6. Examination of Foam Cells Formation in Monocyte-Derived Macrophages Treated with L1 or L5 

To induce differentiation of human monocytes into macrophages, THP-1 cells (1 × 10^5^ cells/mL) were grown in media and treated with 10 ng/mL PMA (MilliporeSigma, Temecula, CA, USA) for 48 h. The culture medium was subsequently changed to RPMI and 1% lipid-depleted fetal bovine serum (FBS) from differential ultracentrifugation as described previously [43]. Then, the macrophages were treated with different doses of L1 or L5 (10, 25, or 50 μg/mL) at 37 °C for 48 h. Foam cell formation in macrophages was examined by using Oil red O staining [44]. The images for foam cell formation were observed in an ECLIPSE 50i microscope (Nikon, Tokyo, Japan) and captured at 100× with NIS-Elements software (Nikon, Tokyo, Japan). The percentage of foam cell formation was quantitated by dividing the number of Oil red O staining macrophages by the total number of macrophages in 2 random microscopic fields.

### 4.7. Database Search, RNA Extraction, and Quantitative Real-Time PCR for Their mRNA Expression Levels

To identify the potential genes involved in the pathogenesis of atherosclerosis in RA patients, we searched the NCBI Gene Expression Omnibus (GEO) database. In group 1, which includes genes associated with hyperlipidemia or CVD, we download 250 genes with the highest (>2) fold change from GSE6054 (Monocytes of patients with familial hypercholesterolemia show alterations in cholesterol metabolism) and GSE62646 (Altered gene expression pattern in peripheral blood mononuclear cells in patients with acute myocardial infarction) through the analysis tool GE02R. In group 2, which includes differentially expressed genes from RA patients compared to healthy controls, we download 250 genes with the highest (>2) fold change from GSE56649 (Expression data from active rheumatoid arthritis patients and healthy control), GSE64707 (Gene expression of human peripheral blood cells of patients with rheumatoid arthritis), and GSE93777 (Multi-omics monitoring of drug response in rheumatoid arthritis). After an integrated analysis, we identified 20 overlapping genes from both groups as target genes for examining mRNA expression using qRT-PCR. The primer sequences of the selected genes were searched from PrimerBank-MGH-PGA (https://pga.mgh.harvard.edu/primerbank/) and are listed in Table S1. Primers were designed and synthesized by Tri-I Biotech (Taipei, Taiwan).

PBMCs were isolated using the Ficoll-Paque^TM^ PLUS (GE Healthcare Biosciences, Uppsala, Sweden) density gradient centrifugation. Total RNAs from PBMCs were extracted by TRI Reagent (Sigma-Aldrich, Missouri, USA) according to the manufacturer’s instructions. A High-Capacity cDNA Reverse Transcriptase Kit (Thermo Fisher Scientific) was used to reverse-transcribe 2 μg RNA into cDNA used for qRT-PCR analyses. The qRT-PCR reactions were performed on the CFX96 Real-time PCR system (Bio-Rad) with IQ^TM^ SYBR Green Supermix reagent (Bio-Rad). Quantitative real-time PCR using 10 ng cDNA was performed with one cycle of preincubation at 95 °C for 3 min, 45 cycles of amplification (95 °C for 15 s, 60 °C for 1 min), and the melt curve detection program from 55 °C to 95 °C. The difference in expression in the target gene relative to the averaged internal control gene was calculated by 2^−△Ct^, △Ct = Ct_targeted genes_−Ct_Actin_.

We observed a significant difference in the mRNA expression levels of 10 targeted genes between RA patients and healthy controls. Subsequently, we evaluated the difference in mRNA expression levels in the 10 candidate genes between RA patients with a high L5% and with normal L5%. The results showed a significant difference in the mRNA expression levels of one gene, *ITGAX*, between the two groups. Then, we examined the mRNA expression levels of CD11c in 93 RA patients and 41 healthy controls. The primer sequences are as follows: For CD11c (*ITGAX*), 5′-CTGCAA GGGTTTACATACACGG-3′ (forward) and 5′-GAATTTTGGCGGCATCCCTAC-3′ (reverse); and for the housekeeping gene, *Actin*, 5′-ATTGCCGACAGGATGCAGA-3′ (forward) and 5′-GAGTACTTGCGCTCAGGAGGA-3′ (reverse). To standardize mRNA expression levels of CD11c, the mRNA levels of actin were also determined in parallel for each sample. The mRNA expression levels of CD11c were calculated using the comparative threshold cycle (Ct) method and evaluated by 2^−^**^△^**^Ct^, △Ct = Ct_CD11c_–Ct_Actin_.

### 4.8. Determination of CD11c Expression in THP-1 Cells Treated with Different Doses of L5 by Flow Cytometry Analysis

The human monocytic cell line, THP-1 cells (ATCC TIB-202; American Type Culture Collection, Manassas, VA, USA), was grown in RPMI 1640 (Thermo Fisher Scientific, Taichung, Taiwan) supplemented with 10% FBS and 1% penicillin/streptomycin antibiotics in an incubator (Thermo Fisher Scientific GmbH, Dreieich, Germany) containing 5% CO_2_ at 37 °C. At the beginning of the lipid treatment experiment, the medium was changed to RPMI with 1% lipid-depleted FBS. THP-1 cells, at a density of 1 × 10^5^ cells/mL, were treated with L5 (10 or 50 μg/mL) at 37 °C for 2 days. The cells were harvested and washed with phosphate-buffered saline (PBS) and then blocked with TruStain FcX (BioLegend, San Diego, CA, USA) at room temperature for 10 min. The CD11c levels of THP-1 samples were quantified by using FITC (fluorescein isothiocyanate)-conjugated anti-CD11c antibody (BD Biosciences Pharmingen, San Diego, CA, USA) and flow cytometry FACSCelesta™ (BD Biosciences) according to the manufacturer’s protocol and the described technique [45]. FITC-conjugated IgG isotype antibody (BD Biosciences) (4 °C, 30 min) served as isotype controls. We analyzed at least 10,000 cells/condition in duplicate. Data analysis was performed with FlowJo software (BD Biosciences). We gain major cell populations (named G1) with dot plots (FSC:SSC) by using an unstained control. Data were expressed as the frequency of CD11c in the gated cell population

### 4.9. Measurement of Plasma Levels of Inflammatory Mediators

Plasma levels of IL-6, IL-8, TNF-α, interferon gamma-induced protein 10 (IP-10), monocyte chemoattractant protein-1 (MCP-1), granulocyte-macrophage colony-stimulating factor (GM-CSF), IL-10, IL-12p40, soluble CD40 ligand (sCD40L), IL-1Ra, and IL-1β were determined using the HCYTOMAG-60K Cytokine/Chemokine Panel assay according to the manufacturer’s instruction (MilliporeSigma, Waltham, MA, USA). Data were analyzed with five-parameter logistic regression by using the MILLIPLEX® Analyst (MilliporeSigma, Burlington, NJ, USA). The overall intra-assay and inter-assay coefficients of variability were calculated (<5% and 20%, respectively).

### 4.10. Measurement of mRNA Expression Levels of Inflammatory Mediators

To investigate the cytokines mRNA expression of L5-treated monocyte/macrophage, we extracted RNA by the TRI Reagent method (Sigma-Aldrich). The High-Capacity cDNA Reverse Transcriptase Kit (ThermoFisher) was used to reverse-transcribe 1 μg RNA into cDNA using for qRT-PCR analyses. The expression levels of each gene were determined by the CFX96 Real-time PCR system (BioRad, Hercules, USA) with IQ SYBR Green Supermix reagent (BioRad). PCR using 10 ng cDNA was performed with one cycle of preincubation at 95 °C for 3 min, 45 cycles of amplification (95 °C for 15 s, 60 °C for 1 min), and the melt curve detection program from 55 °C to 95 °C. The primer sequences are as follows: IL-6, 5′-AGACAGCCACTCACCTCTTCAG-3′ (forward) and 5′-TTCTGCCAGTGCCTCTTTGCTG-3′ (reverse); IL-8, 5′-GAGAGTGATTGAGAGTGGACCAC-3′ (forward) and 5′-CACAACCCTCTGCACCCAGTTT-3′ (reverse); TNF-α, 5′-CCACTTCGAAACCTGGGATTC-3′ (forward) and 5′-TTAGTGGTTGCCAGCACTTCA-3′ (reverse). The mRNA expression levels of cytokines were calculated using the comparative threshold cycle (Ct) method and were evaluated by 2^−△Ct^, △Ct = Ct_Target gene_–Ct_Actin_. The results were normalized to the levels of actin mRNA and were expressed relative to the levels in control cells (relative value = 1).

### 4.11. Statistical Analysis

The results are presented as the mean ± standard deviation (SD), the standard error of mean (SEM), or the median (interquartile range). The nonparametric Mann−Whitney U test was used for between-group comparisons of plasma levels of lipid profile, AI, and QRISK-2 scores. The comparison of mRNA expression levels between RA patients and healthy controls or between RA patients with high L5% and normal L5% was analyzed by the Student’s t-test. The comparison of CD11c expression or cytokine mRNA expression levels in THP-1 cells or macrophages treated with different doses of L1 and L5 was analyzed by one-way ANOVA. The correlation coefficient was calculated using the nonparametric Spearman’s rank correlation test. A two-sided *p*-value < 0.05 was considered statistically significant.

## 5. Conclusions

L5 may contribute to atherosclerosis by augmenting macrophage foam cell formation, upregulating CD11c expression, or enhancing the expression of inflammatory mediators, such as IL-6, IL-8, and TNF-α. These findings provide new insight into the pathogenesis of increased CVD risk in RA that cannot be explained by conventional risk factors.

## Figures and Tables

**Table 1:** Demographic and laboratory data in rheumatoid arthritis (RA) patients with high percentage of L5 in low-density lipoprotein (LDL) (L5%) and with normal L5% **^a.^**

	RA with High L5% (*n* = 30)	RA with Normal L5% (*n* = 63)
Age at entry, years	60.4 ± 10.9	58.4 ± 12.1
Women proportion	25 (83.3%)	50 (79.4%)
RA duration, months	68.9 ± 22.6	74.9 ± 28.4
BMI, kg/m^2^	23.7 ± 2.2	23.0 ± 2.3
RF positivity	20 (66.7%)	46 (73.0%)
ACPA positivity	18 (60.0%)	45 (71.4%)
ESR, mm/1^st^ hour	24.9 ± 12.2	20.4 ± 15.6
CRP, mg/dl	1.08 ± 1.07 ^b^	0.61 ± 0.69
DAS28 at study entry	4.25 ± 1.27	3.78 ± 1.07
Daily steroid dose (mg)	4.8 ± 1.7	4.2 ± 2.0
csDMARDs alone at entry	8 (26.7%)	15 (23.8%)
Biologics used at entry		
TNF-α inhibitors	11 (36.7%)	19 (30.2%)
IL-6R inhibitor	9 (30.0%)	17 (27.0%)
Rituximab	2 (6.7%)	2 (3.2%)
Hypertension	12 (40.0%)	20 (31.7%)
Diabetes mellitus	5 (16.7%)	6 (9.5%)
Current smoker	2 (6.7%)	5 (7.9%)
TC, mg/dl	207 (164-236)	211 (176–244)
HDL-C, mg/dl	59.5 (44.5–74.8)	59.6 (49.4–75.0)
Triglyceride, mg/dl	114 (77–151)	100 (67–138)
LDL-C, mg/dl	129 (87–154)	129 (105–154)
Atherogenic index	3.4 (2.5–4.4)	3.3 (2.7–4.3)
QRISK-2 score	8.7 (5.8–14.5) ^c^	5.7 (2.7–9.1)
CVD events	6 (20.0%) ^d^	4 (6.3%) ^e^

^a^ Data are presented as median (interquartile range, IQR), mean ± standard deviation (SD), or number (%). High L5% is defined as plasma L5 proportion above 1.8%. ^b^ *p* < 0.05 and ^c^ *p* < 0.01 vs. patients with normal L5%, as determined by using the Mann–Whitney U test. ^d^ Included two patients with acute ST-segment elevation myocardial infarction and four with ischemic stroke; ^e^ included two patients with acute ST-segment elevation myocardial infarction and two with ischemic stroke; BMI: Body mass index; ACPA: Anti-citrullinated peptide antibodies; ESR: Erythrocyte sedimentation rate; CRP: C-reactive protein; DAS28: Disease activity score for 28-joints; csDMARDs: Conventional synthetic disease-modifying anti-rheumatic drugs; TNF-α: Tumor necrosis factor-α; IL-6: Interleukin-6; TC: Total cholesterol; HDL-C: High-density lipoprotein cholesterol; LDL-C: Low-density lipoprotein cholesterol; atherogenic index is the ratio of TC/HDL-C; CVD: Cerebrovascular/cardiovascular disease.

## Supplementary Materials

Supplementary materials can be found at https://www.mdpi.com/1422-0067/21/16/5883/s1.

## Abbreviations

ABCA1	ATP-binding cassette, sub-family A, member 1
ACPA	Anti-citrullinated peptide antibodies
AI	Atherogenic index
Apo	Apolipoprotein
CRP	C-reactive protein
csDMARD	conventional synthetic disease-modifying anti-rheumatic drug
CVD	Cardiovascular/cerebrovascular disease
DAS28	28-joint disease activity score
GEO	Gene Expression Omnibus
GM-CSF	Granulocyte-macrophage colony-stimulating factor
HC	Healthy controls
HDL-C	High-density lipoprotein cholesterol
IL	Interleukin
IP-10	Interferon gamma-induced protein 10
ITGAX	Integrin Subunit Alpha X
L5%	Percentage of L5 in LDL
LDL	Low-density lipoprotein
LDL-C	Low-density lipoprotein cholesterol
MCP-1	Monocyte chemoattractant protein-1
NPC1	Niemann-Pick disease, type C1
PMA	Phorbol myristate acetate
qRT-PCR	Quantitative real-time polymerase chain reaction
RA	Rheumatoid arthritis
RF	Rheumatoid factor
sCD40L	Soluble CD40 ligand
SD	Standard deviation
SEM	Standard error of mean
SLE	Systemic lupus erythematosus
TC	Total cholesterol
TNF-α	Tumor necrosis factor-α

## References

[1] Libby P. (2012). Inflammation in atherosclerosis. Arter. Thromb. Vasc. Biol..

[2] Cvetkovic J.T., Wallberg-Jonsson S., Ahmed E., Rantapaa-Dahlqvist S., Lefvert A.K. (2002). Increased levels of autoantibodies against copper-oxidized low density lipoprotein, malondialdehyde-modified low density lipoprotein and cardiolipin in patients with rheumatoid arthritis. Rheumatology.

[3] Choy E.H., Panayi G.S. (2001). Cytokine pathways and joint inflammation in rheumatoid arthritis. N. Engl. J. Med..

[4] Avina-Zubieta J.A., Thomas J., Sadatsafavi M., Lehman A.J., Lacaille D. (2012). Risk of incident cardiovascular events in patients with rheumatoid arthritis: A meta-analysis of observational studies. Ann. Rheum. Dis..

[5] Symmons D.P., Gabriel S.E. (2011). Epidemiology of CVD in rheumatic disease, with a focus on RA and SLE. Nat. Rev. Rheumatol..

[6] Im C.H., Kim N.R., Kang J.W., Kim J.H., Kang J.Y., Bae G.B., Nam E.J., Kang Y.M. (2015). Inflammatory burden interacts with conventional cardiovascular risk factors for carotid plaque formation in rheumatoid arthritis. Rheumatology.

[7] Choy E., Ganeshalingam K., Semb A.G., Szekanecz Z., Nurmohamed M. (2014). Cardiovascular risk in rheumatoid arthritis: Recent advances in the understanding of the pivotal role of inflammation, risk predictors and the impact of treatment. Rheumatology.

[8] Bacchiega B.C., Bacchiega A.B., Usnayo M.J., Bedirian R., Singh G., Pinheiro G.D. (2017). Interleukin 6 Inhibition and Coronary Artery Disease in a High-Risk Population: A Prospective Community-Based Clinical Study. J. Am. Heart Assoc..

[9] Rho Y.H., Chung C.P., Oeser A., Solus J., Asanuma Y., Sokka T., Pincus T., Raggi P., Gebretsadik T., Shintani A. (2009). Inflammatory mediators and premature coronary atherosclerosis in rheumatoid arthritis. Arthritis Rheum..

[10] Boisvert W.A., Curtiss L.K., Terkeltaub R.A. (2000). Interleukin-8 and its receptor CXCR2 in atherosclerosis. Immunol. Res..

[11] Barnabe C., Martin B.J., Ghali W.A. (2011). Systematic review and meta-analysis: Anti-tumor necrosis factor alpha therapy and cardiovascular events in rheumatoid arthritis. Arthritis Care Res..

[12] Gonzalez-Juanatey C., Vazquez-Rodriguez T.R., Miranda-Filloy J.A., Gomez-Acebo I., Testa A., Garcia-Porrua C., Sanchez-Andrade A., Llorca J., Gonzalez-Gay M.A. (2012). Anti-TNF-alpha-adalimumab therapy is associated with persistent improvement of endothelial function without progression of carotid intima-media wall thickness in patients with rheumatoid arthritis refractory to conventional therapy. Mediat. Inflamm..

[13] Myasoedova E., Crowson C.S., Kremers H.M., Roger V.L., Fitz-Gibbon P.D., Therneau T.M., Gabriel S.E. (2011). Lipid paradox in rheumatoid arthritis: The impact of serum lipid measures and systemic inflammation on the risk of cardiovascular disease. Ann. Rheum. Dis..

[14] Niccoli G., Baca M., De Spirito M., Parasassi T., Cosentino N., Greco G., Conte M., Montone R.A., Arcovito G., Crea F. (2012). Impact of electronegative low-density lipoprotein on angiographic coronary atherosclerotic burden. Atherosclerosis.

[15] Chang C.Y., Chen C.H., Chen Y.M., Hsieh T.Y., Li J.P., Shen M.Y., Lan J.L., Chen D.Y. (2019). Association between Negatively Charged Low-Density Lipoprotein L5 and Subclinical Atherosclerosis in Rheumatoid Arthritis Patients. J. Clin. Med..

[16] Chan H.C., Chan H.C., Liang C.J., Lee H.C., Su H., Lee A.S., Shiea J., Tsai W.C., Ou T.T., Wu C.C. (2020). Role of Low-Density Lipoprotein in Early Vascular Aging Associated with Systemic Lupus Erythematosus. Arthritis Rheumatol..

[17] Kathiresan S., Srivastava D. (2012). Genetics of human cardiovascular disease. Cell.

[18] Abbate R., Sticchi E., Fatini C. (2008). Genetics of cardiovascular disease. Clin. Cases Min. Bone Metab..

[19] Chistiakov D.A., Melnichenko A.A., Myasoedova V.A., Grechko A.V., Orekhov A.N. (2017). Mechanisms of foam cell formation in atherosclerosis. J. Mol. Med..

[20] Yu X.H., Jiang N., Yao P.B., Zheng X.L., Cayabyab F.S., Tang C.K. (2014). NPC1, intracellular cholesterol trafficking and atherosclerosis. Clin. Chim. Acta.

[21] Kurohori Y., Sato K., Suzuki S., Kashiwazaki S. (1995). Adhesion molecule expression on peripheral blood mononuclear cells in rheumatoid arthritis: Positive correlation between the proportion of L-selectin and disease activity. Clin. Rheumatol..

[22] Sandor N., Lukacsi S., Ungai-Salanki R., Orgovan N., Szabo B., Horvath R., Erdei A., Bajtay Z. (2016). CD11c/CD18 Dominates Adhesion of Human Monocytes, Macrophages and Dendritic Cells over CD11b/CD18. PLoS ONE.

[23] Wu H., Gower R.M., Wang H., Perrard X.Y., Ma R., Bullard D.C., Burns A.R., Paul A., Smith C.W., Simon S.I. (2009). Functional role of CD11c+ monocytes in atherogenesis associated with hypercholesterolemia. Circulation.

[24] Vianello E., Dozio E., Arnaboldi F., Marazzi M.G., Martinelli C., Lamont J., Tacchini L., Sigruner A., Schmitz G., Corsi Romanelli M.M. (2016). Epicardial adipocyte hypertrophy: Association with M1-polarization and toll-like receptor pathways in coronary artery disease patients. Nutr. Metab. Cardiovasc. Dis..

[25] Shu Q.H., Ge Y.S., Ma H.X., Gao X.Q., Pan J.J., Liu D., Xu G.L., Ma J.L., Jia W.D. (2016). Prognostic value of polarized macrophages in patients with hepatocellular carcinoma after curative resection. J. Cell. Mol. Med..

[26] Shapouri-Moghaddam A., Mohammadian S., Vazini H., Taghadosi M., Esmaeili S.A., Mardani F., Seifi B., Mohammadi A., Afshari J.T., Sahebkar A. (2018). Macrophage plasticity, polarization, and function in health and disease. J. Cell. Physiol..

[27] Cho K.Y., Miyoshi H., Kuroda S., Yasuda H., Kamiyama K., Nakagawara J., Takigami M., Kondo T., Atsumi T. (2013). The phenotype of infiltrating macrophages influences arteriosclerotic plaque vulnerability in the carotid artery. J. Stroke Cereb. Dis..

[28] Mahmoudi M., Aslani S., Fadaei R., Jamshidi A.R. (2017). New insights to the mechanisms underlying atherosclerosis in rheumatoid arthritis. Int. J. Rheum. Dis..

[29] Wen W., He M., Liang X., Gao S.S., Zhou J., Yuan Z.Y. (2016). Accelerated transformation of macrophage-derived foam cells in the presence of collagen-induced arthritis mice serum is associated with dyslipidemia. Autoimmunity.

[30] Lee A.S., Wang Y.C., Chang S.S., Lo P.H., Chang C.M., Lu J., Burns A.R., Chen C.H., Kakino A., Sawamura T. (2020). Detection of a High Ratio of Soluble to Membrane-Bound LOX-1 in Aspirated Coronary Thrombi From Patients With ST-Segment-Elevation Myocardial Infarction. J. Am. Heart Assoc..

[31] Ke L.Y., Chan H.C., Chen C.C., Chang C.F., Lu P.L., Chu C.S., Lai W.T., Shin S.J., Liu F.T., Chen C.H. (2020). Increased APOE glycosylation plays a key role in the atherogenicity of L5 low-density lipoprotein. FASEB J..

[32] Kawakami A., Aikawa M., Libby P., Alcaide P., Luscinskas F.W., Sacks F.M. (2006). Apolipoprotein CIII in apolipoprotein B lipoproteins enhances the adhesion of human monocytic cells to endothelial cells. Circulation.

[33] Chu C.S., Chan H.C., Tsai M.H., Stancel N., Lee H.C., Cheng K.H., Tung Y.C., Chan H.C., Wang C.Y., Shin S.J. (2018). Range of L5 LDL levels in healthy adults and L5’s predictive power in patients with hyperlipidemia or coronary artery disease. Sci. Rep..

[34] Shen M.Y., Chen F.Y., Hsu J.F., Fu R.H., Chang C.M., Chang C.T., Liu C.H., Wu J.R., Lee A.S., Chan H.C. (2016). Plasma L5 levels are elevated in ischemic stroke patients and enhance platelet aggregation. Blood.

[35] Arida A., Protogerou A.D., Kitas G.D., Sfikakis P.P. (2018). Systemic Inflammatory Response and Atherosclerosis: The Paradigm of Chronic Inflammatory Rheumatic Diseases. Int. J. Mol. Sci..

[36] Bancells C., Canals F., Benitez S., Colome N., Julve J., Ordonez-Llanos J., Sanchez-Quesada J.L. (2010). Proteomic analysis of electronegative low-density lipoprotein. J. Lipid. Res..

[37] Chistiakov D.A., Bobryshev Y.V., Orekhov A.N. (2015). Changes in transcriptome of macrophages in atherosclerosis. J. Cell. Mol. Med..

[38] Charles-Schoeman C., Gonzalez-Gay M.A., Kaplan I., Boy M., Geier J., Luo Z., Zuckerman A., Riese R. (2016). Effects of tofacitinib and other DMARDs on lipid profiles in rheumatoid arthritis: Implications for the rheumatologist. Semin. Arthritis Rheum..

[39] Aletaha D., Neogi T., Silman A.J., Funovits J., Felson D.T., Bingham C.O., Birnbaum N.S., Burmester G.R., Bykerk V.P., Cohen M.D. (2010). Rheumatoid arthritis classification criteria: An American College of Rheumatology/European League Against Rheumatism collaborative initiative. Ann. Rheum. Dis..

[40] Prevoo M.L., Van ’t Hof M.A., Kuper H.H., Van Leeuwen M.A., Van de Putte L.B., Van Riel P.L. (1995). Modified disease activity scores that include twenty-eight-joint counts. Development and validation in a prospective longitudinal study of patients with rheumatoid arthritis. Arthritis Rheum..

[41] Hippisley-Cox J., Coupland C., Vinogradova Y., Robson J., Minhas R., Sheikh A., Brindle P. (2008). Predicting cardiovascular risk in England and Wales: Prospective derivation and validation of QRISK2. BMJ.

[42] Ke L.Y., Chan H.C., Chan H.C., Kalu F.C.U., Lee H.C., Lin I.L., Jhuo S.J., Lai W.T., Tsao C.R., Sawamura T. (2017). Electronegative Low-Density Lipoprotein L5 Induces Adipose Tissue Inflammation Associated With Metabolic Syndrome. J. Clin. Endocrinol. Metab..

[43] Havel R.J., Eder H.A., Bragdon J.H. (1955). The distribution and chemical composition of ultracentrifugally separated lipoproteins in human serum. J. Clin. Investig..

[44] Halvorsen B., Waehre T., Scholz H., Clausen O.P., Von der Thusen J.H., Muller F., Heimli H., Tonstad S., Hall C., Froland S.S. (2005). Interleukin-10 enhances the oxidized LDL-induced foam cell formation of macrophages by antiapoptotic mechanisms. J. Lipid. Res..

[45] Scott C.S., Richards S.J., Master P.S., Kendall J., Limbert H.J., Roberts B.E. (1990). Flow cytometric analysis of membrane CD11b, CD11c and CD14 expression in acute myeloid leukaemia: Relationships with monocytic subtypes and the concept of relative antigen expression. Eur. J. Haematol..

