# Supplementary material for: Association of Electronegative LDL with Macrophage Foam Cell Formation and CD11c Expression in Rheumatoid Arthritis Patients
Source: Int J Mol Sci. 2020 Aug 16;21(16):5883. doi: 10.3390/ijms21165883 (PMC7461586; doi:10.3390/ijms21165883)
Supplement: Supplementary file 1 [file ijms-21-05883-s001.pdf]

Table S1. The primer list used in qRT-PCR.

Gene Name	Gene ID	Primer	Sequence
Niemann-Pick disease, type C1 (NPC1)	4864	F	GCACCTTTTACCATCACTCCTG
		R	GGCCACAGACAATAGAGCAGT
ATP-binding cassette, sub-family A (ABC1), member 1 (ABCA1)	19	F	ACCCACCCTATGAACAACATGA
		R	GAGTCGGGTAACGGAAACAGG
trinucleotide repeat containing 6A (TNRC6A)	27327	F	AGCCACAAAATAACGGAGAGG
		R	CGACAAGTCACTTACAACAGCAT
SWI/SNF related, matrix associated, actin dependent regulator of chromatin, subfamily a, member 2 (SMARCA2)	6595	F	CACAGCCAAGGTTATATGTCACC
		R	TGCGGATCACCTGGGATGA
zinc finger protein 652 (ZNF652)	22834	F	AGAGAAAGTCAGCGTTACACAAA
		R	CGACCTCTTGTAGTTCTGGGAG
ARP2 actin-related protein 2 homolog (ACTR2)	10097	F	GGCAGTTCTGACTTTGTACGC
		R	CCAGTCTCCTGGTAAGATGAGG
protein tyrosine phosphatase, receptor type, f polypeptide (PTPRF), interacting protein (liprin), alpha 1 (PPFIA1)	8500	F	AAAAGGTGAGAGAGCGATTACG
		R	TGTCCAGCACTCCATCTGTTA
keratin 10 (KRT10)	3858	F	TCCTACTTGGACAAAGTTCGGG
		R	CCCCTGATGTGAGTTGCCA
WD repeat and SOCS box containing 1 (WSB1)	26118	F	GGTGTCAGCTTCAAGAGACAAA
		R	AGTCAGGAGAGAATGCACAGC
fibrosin-like 1 (FBRSL1)	57666	F	CCCCTACTTCCGACATTCCAG
		R	AGACACCTTAGGCTGAAAAGC
lysosomal trafficking regulator (LYST)	1130	F	GCAACAGATTTTAACCTACCGCT
		R	AACCTGACTAGACAGGGCACT
SUMO1/sentrin specific peptidase 7 (SENP7)	57337	F	GTTGACGAATGTCCTATGGACG
		R	AATGAGTCTGATTGCACCTTGTT
heterogeneous nuclear ribonucleoprotein A2/B1 (HNRNPA2B1)	3181	F	ATTGATGGGAGAGTAGTTGAGCC
		R	AATTCCGCCAACAAACAGCTT
U2AF homology motif (UHM) kinase 1 (UHMK1)	127933	F	ACGCTGTCTGTTGCTTGAACT
		R	GGCACAATGCTGTATCATCCAC
solute carrier family 25 (mitochondrial carrier; adenine	293	F	GCAACCTTGCCAACGTCATTC
		R	CCGCAAAGTACCTCCAGAACT

**nucleotide translocator), member 6 (SLC25A6)**			
**integrin, alpha X (ITGAX)**	**3687**	**F**	**CTGCAAGGGTTTACATACACGG**
		**R**	**GAATTTTGGCGGCATCCCTAC**
**eukaryotic translation initiation factor 5 (EIF5)**	**1983**	**F**	**TTAATCGGCCTCCAACGTATCC**
		**R**	**CTTGCAGCTTATTCGCCTCAT**
**AF4/FMR2 family, member 4 (AFF4)**	**27125**	**F**	**ACCGTGAAGACCGGAATGTG**
		**R**	**GAGAGGAGAGCTAGGTGGGAA**
**peroxisome proliferator-activated receptor gamma, coactivator-related 1 (PPRC1)**	**23082**	**F**	**GAGCAGGTTATCTCTGGAGGA**
		**R**	**AGCAGCTCCGAATCAGGAATG**
**zinc finger, AN1-type domain 6 (ZFAND6)**	**54469**	**F**	**AAGTGCCTATGCTTTGTTCCAC**
		**R**	**ATGCCATTTGTACGAGGGTTT**

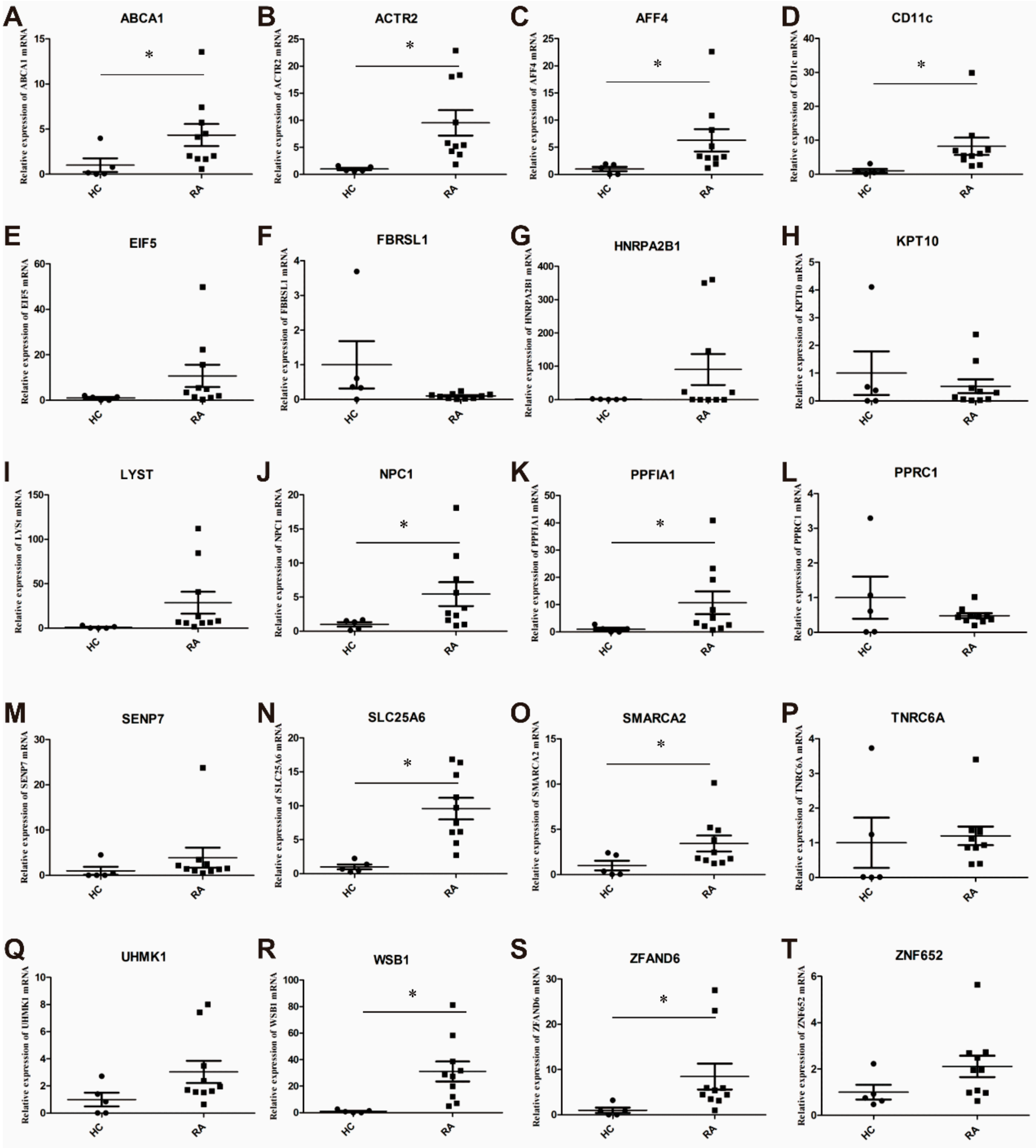

Figure S1. The mRNA expression levels between RA patients and with health control (HC) in pilot study. Value are the mean ± SEM. *p<0.05.

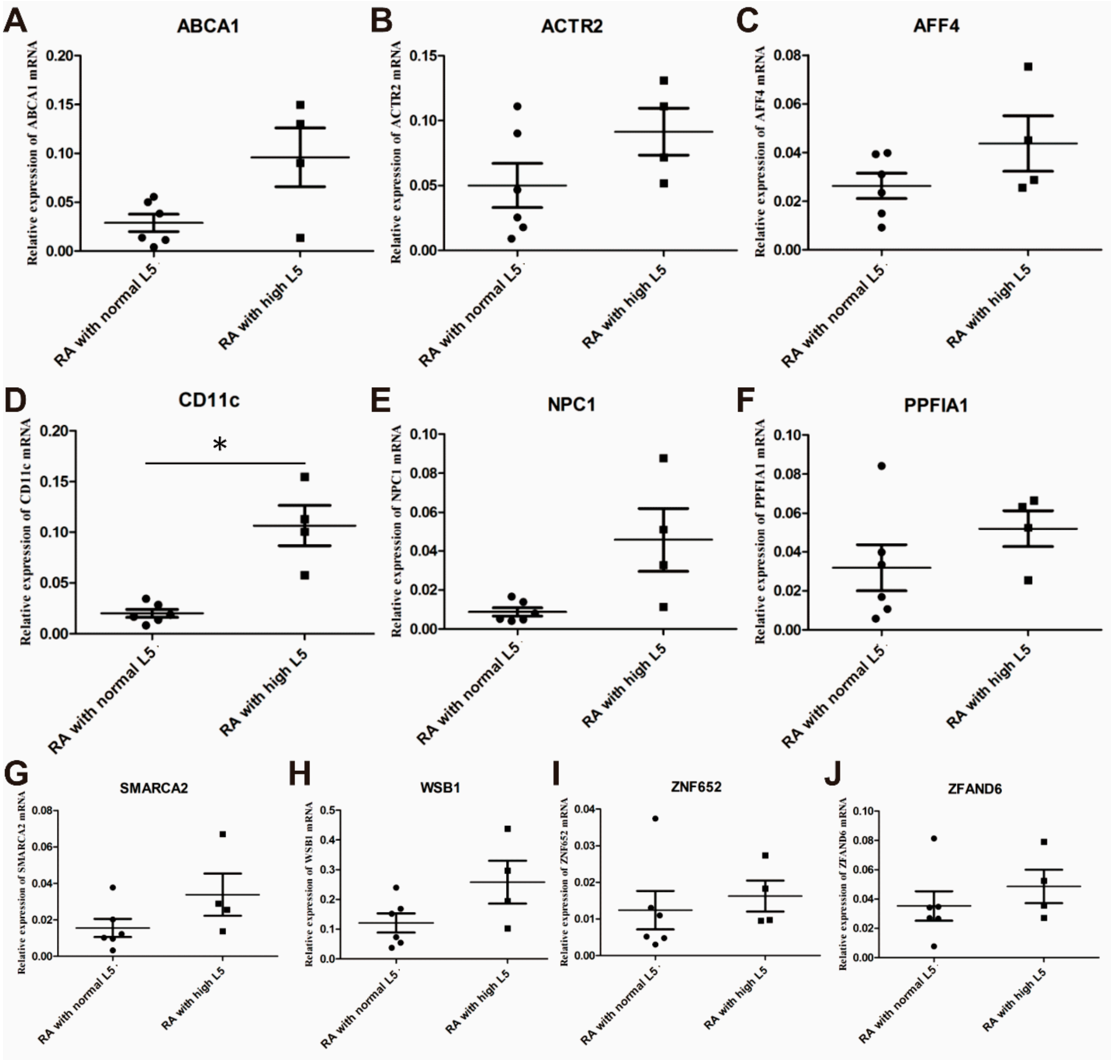

Figure S2. The mRNA expression levels between RA patients with high L5% and with normal L5% in pilot study. Value are the mean ± SEM. *p<0.05.

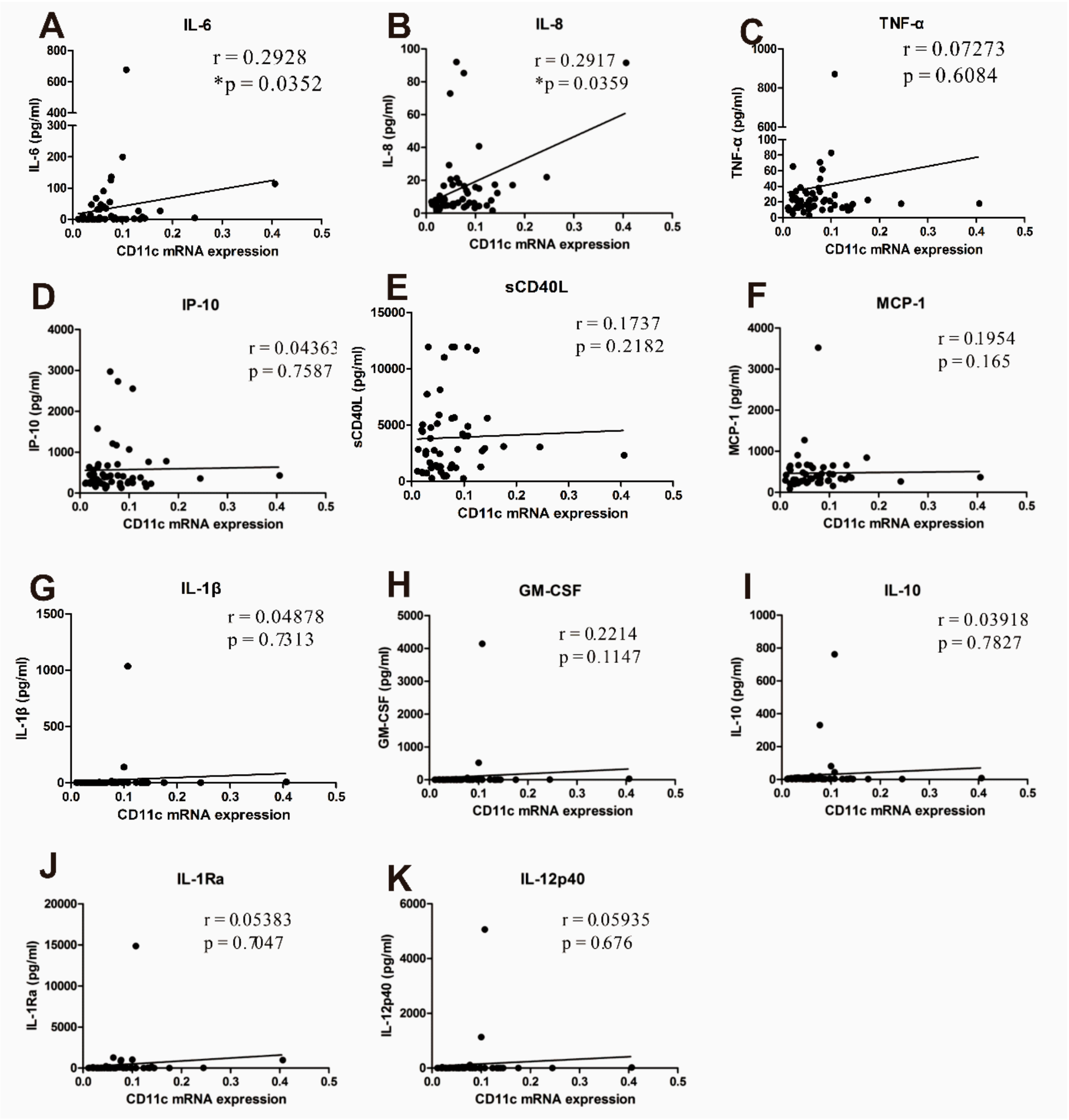

Figure S3. Correlation between the CD11c expression and plasma levels of inflammatory mediators in RA patients. The correlation coefficient was calculated using the nonparametric Spearman's rank correlation test. *p<0.05.
